# Papulopustular Dermatitis in X-Linked Chronic Granulomatous Disease

**DOI:** 10.3389/fped.2018.00429

**Published:** 2019-01-31

**Authors:** Puja Sood Rajani, Maria A. Slack

**Affiliations:** Division of Pediatric Allergy and Immunology, Department of Pediatrics, University of Rochester Medical Center, Golisano Children's Hospital, Rochester, NY, United States

**Keywords:** rash, papulopustlar lesions, chronic granulomatous disease (CGD), immune deficiency, immunodeficiency-primary, dermatitis, papulopustular

## Abstract

Here we describe two term male infants diagnosed with X-linked CGD who present, in addition to frequent infection, with a unique papulopustular skin rash. CGD is caused by a number of genetic defects that impair phagocyte function. This disease results in recurrent infections and granuloma formation. Rarely do patients develop cutaneous symptoms, unless associated with autoimmune disorders such as systemic erythematous lupus (1). Each male infant mentioned here was diagnosed with CGD based on abnormal DHR testing and confirmatory genetic testing. The presenting papulopustular dermatitis was initially characterized as non-classic appearing eczema and subsequently found to be refractory to usual eczema treatment and antibiotics. After obtaining written informed consent from both families, we have documented photographs of the development of a characteristic rash in two newly diagnosed infants with CGD. One infant underwent cutaneous biopsy with histologic evaluation and negative cultures. The dermatitis for both infants was refractory to topical and systemic therapies, and resolved after bone marrow transplantation. Our objective was to characterize cutaneous findings in X-linked CGD and emphasize the importance of considering further immune workup in patients who present with unusual cutaneous findings that do not fit with common infant rashes in conjunction with concerning features for primary immunodeficiency.

## Background

Chronic granulomatous disease (CGD) is caused by genetic defects which impair phagocyte function. This disease results in recurrent infections and granulomatous inflammation. Rarely do patients present with systemic rash as a feature of X-linked CGD, unless associated with autoimmune disorders such as systemic erythematous lupus (1, 2). Previously described cutaneous findings include granulomas, abscesses, photosensitivity, malar rash, discoid lupus, vasculitis, and rarely vesicular rashes due to infection (1–5). Here we describe two term infants diagnosed with X-linked CGD who present, in addition to frequent infection, with a unique papulopustular skin rash initially diagnosed as non-classic appearing eczema refractory to usual eczema treatment and antibiotics.

## Case Presentation

**Figure 1:**
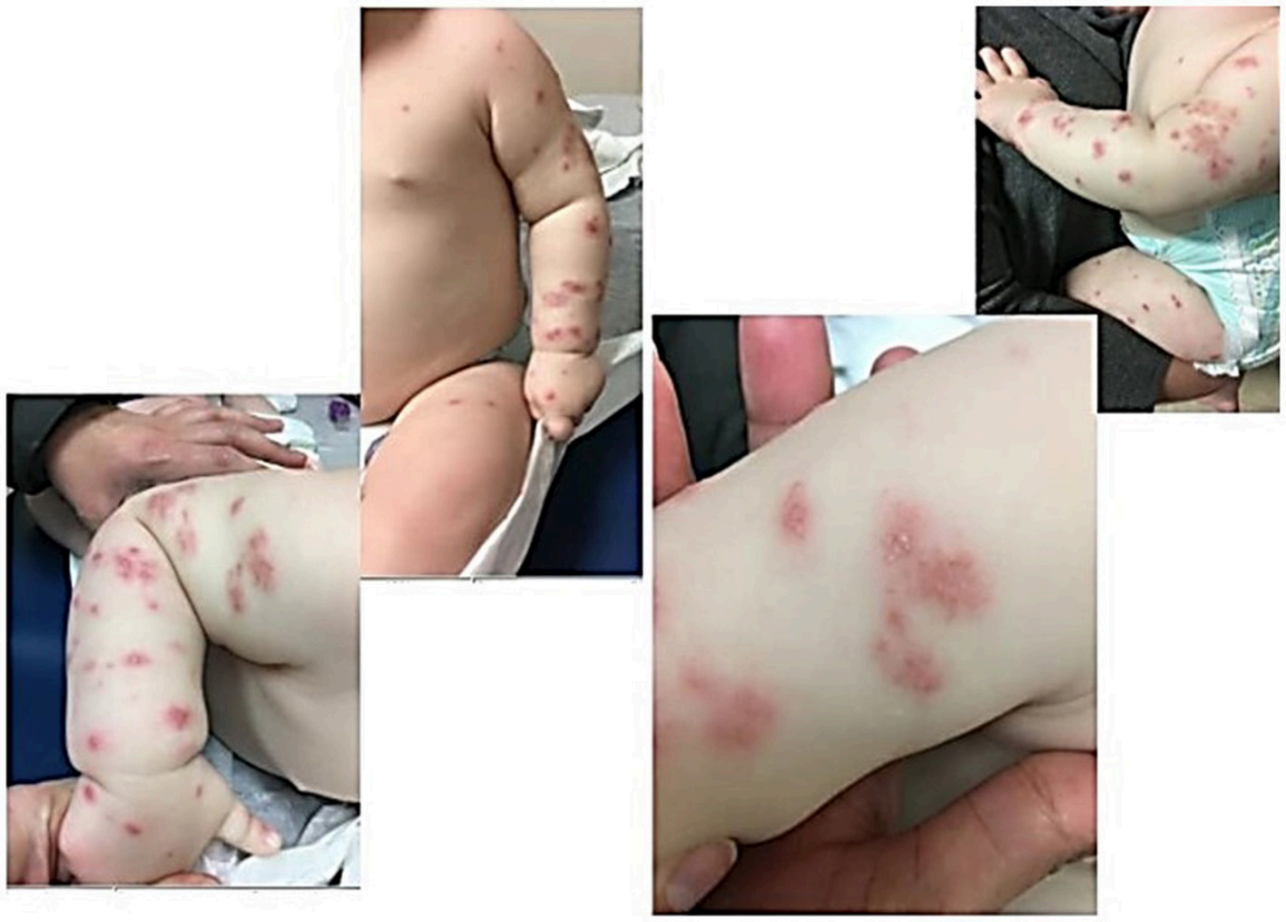
Patient One demonstrates papulopustlar lesions with an erythematous base scattered on upper and lower extremities.

Our first patient presented at 8 months of age with a known history of reflux born full term. His presenting symptoms included episodic fever and chronic leukocytosis, iron deficiency anemia, thrombocytosis, elevated inflammatory markers, blood in the stool concerning for proctocolitis for the past month with elevated calprotectin, and a rash present since 4 months of age. He was on no medications. Lab work showed an elevated WBC to 29.4 × 10^3^/μL (5.0–17.0 × 10^3^/μL), decreased hemoglobin down to 8.6 g/dL (11.5–14.0 g/dL) (thought to be due to blood in the stool), elevated platelet count to 621 × 10^3^/μL (150–330 × 10^3^/μL), ESR max to >130 mm/h, elevated absolute neutrophil count to 13,500 and elevated absolute lymphocyte count to 14,400. Blood cultures were negative, and T, B, and NK lymphocyte subsets were normal. Esophagogastroduodenoscopy with flexible sigmoidoscopy was performed due to his history of reflux and his proctocolitis. It showed no signs of inflammatory bowel disease, the left colon had macroscopically raised erythematous lesions but was microscopically normal with no signs of active colitis. He was initially diagnosed with food protein-inducted enterocolitis syndrome to dairy, given his upper (reflux) and lower (proctocolitis) symptoms, and eczema due to his rash with this allergic profile in the setting of diagnosis of various infections (otitis, upper respiratory illness). The skin rash was described as non-pruritic, generalized pink-purple papulopustular lesions with an erythematous base, most prominent on upper and lower extremities, mostly sparing the trunk (Figure 1). Skin cultures were negative. Skin biopsy was performed and histopathology revealed an essentially unremarkable appearing epidermis and a superficial perivascular lymphohistiocytic infiltrate within the dermis. Eosinophils and plasma cells were not abundant. The histologic changes were thought to be non-specific, but could be seen in a drug reaction, urticaria, or viral exanthem. Initial immunologic evaluation revealed normal levels of IgA, IgG, IgM, IgE and T, B, and NK lymphocyte subsets. Outside of medications and infection, these findings have not been described in CGD related pathology. Giemsa stain and a stain for mast cell tryptase revealed normal numbers of mast cells in the biopsy specimen. He was not on any systemic medications at the time and did not respond to treatment with topical moisturizers, oral antihistamines, topical steroids, dietary avoidances, or any standard for eczema and urticarial care. He had a maternal uncle who passed away in infancy from infection. Heightened clinical suspicion for primary immunodeficiency with this family history and concern that his rash was related to cutaneous inflammatory condition led to a neutrophil oxidative burst assay being obtained. This demonstrated complete loss of neutrophil oxidative function, which was consistent with CGD and genetic testing, thus being confirmatory for X-linked CGD. Pathogenic mutation nucleic acid change was c. 469c>T; Hemizygous; Amino acid alteration was p.157Arg>X within the CYBB gene locus. He had no other features of auto-immune disease; a negative anti-nuclear antibody (ANA) result was obtained later in his clinical course and his proctocolitis was thought to be CGD-associated colitis which is well described in CGD. His rash did not respond to systemic treatments for his colitis, with sulfasalazine, oral antibiotics for both gram positive and gram negative organisms, or IFN-gamma, and he solely improved after hematopoietic stem cell transplantation (HSCT) by day 35 post-transplant. The patient was 18 months of age at the time of transplantation with reduced intensity myeloablative conditioning and has been without recurrence of the rash over the past 18 months. His colitis also improved after transplantation.

**Figure 2:**
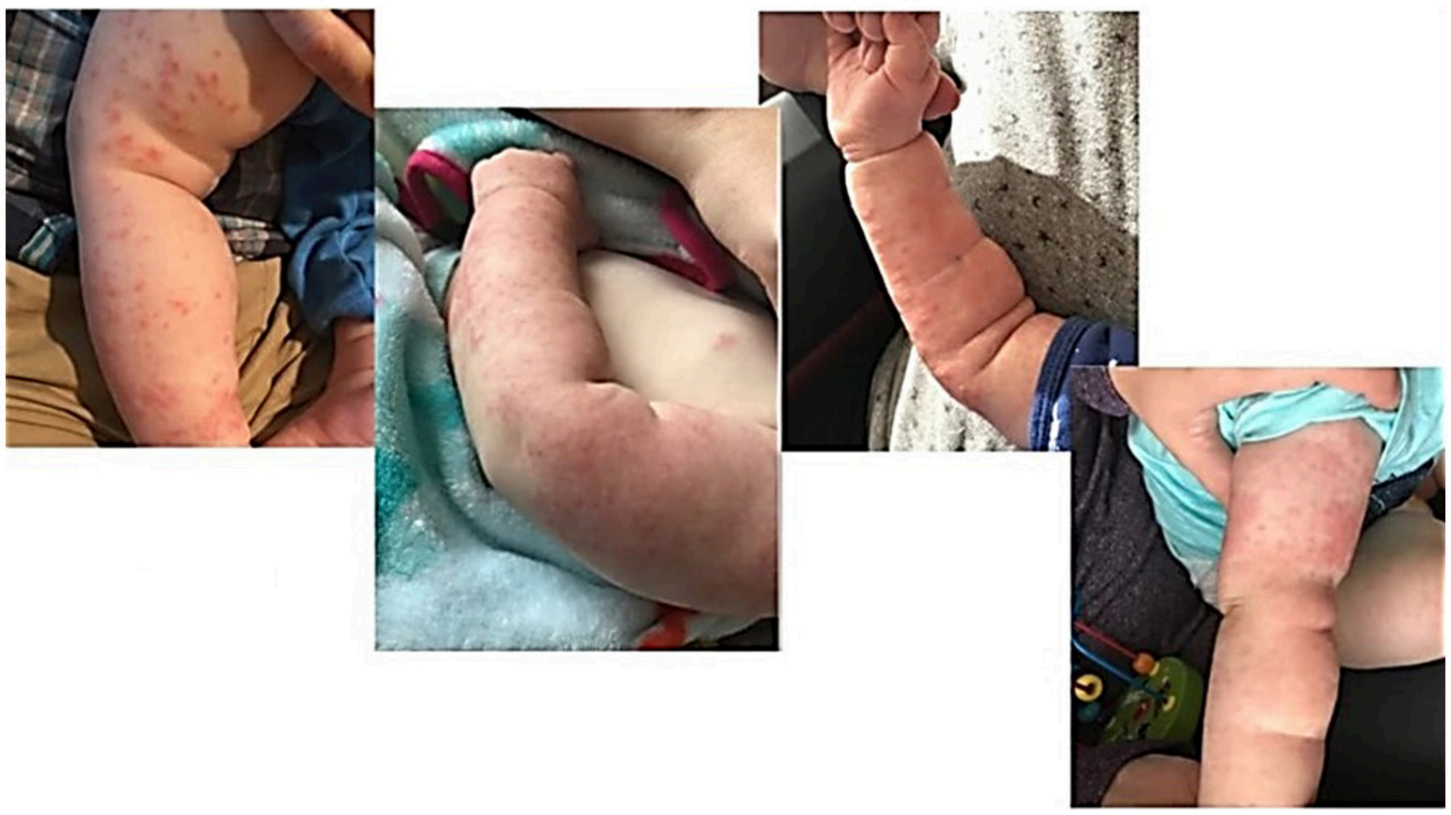
Patient Two demonstrates papulopustular lesions found diffusely scattered on the upper and lower extremities with areas of confluence.

Our second patient presented at 14 months of age with no significant past medical history born full term. He had a paternal history of autoimmune gastrointestinal disease from his father. His presenting symptoms included persistent fevers, inguinal lymphadenopathy, leukocytosis, elevated inflammatory markers, non-bloody diarrhea and rash. He presented to the hospital with a fever of unknown origin with concern for infection, atypical Kawasaki's, or drug rash. His rash was present since 9 months of age and described as scattered, erythematous, papulopustular lesions most prominent on the upper arms but also present on lower arms, legs, and chest, faint lesions on the face (Figure 2). There was some induration and thickness to the rash in some areas (confluent on the arms with more erythema and induration) and more faint pink papules (scattered on legs) elsewhere. Lab work showed an elevated WBC to 20.5 × 10^3^/μL, decreased hemoglobin down to 9.2 g/dL, elevated platelets to 433 × 10^3^/μL, ESR max to 73 mm/h, increased CRP to 104 and elevated ALC to 13,900. Blood cultures were negative, and T, B, and NK subsets were normal. Unfortunately, a skin biopsy of the rash was not performed. Initial differential diagnosis included fever of unknown origin, infection, atypical Kawasaki's, drug rash (he had been on trimethoprim/sulfamethoxazole), eczema flare, malignancy, Kikuchi Fujimoto disease, and tularemia. A biopsy of his inguinal lymph node showed “granulomatous lymphadenitis with neutrophilic abscess formation,” and culture was positive for *Serratia marcescens*, increasing suspicion for CGD. Neutrophil oxidative burst assay showing complete loss of oxidative function was consistent with CGD, and genetic testing was confirmatory for X-linked CGD, due to the presence of a mutation within the CYBB gene. Pathogenic nucleic acid change was c. 141 + 5_G_>_A_; Hemizygous; Amino acid alteration was p.del Exon 2. The rash remained unchanged with treatment for infection, initiation of antifungal and bacterial prophylaxis, along with topical steroid therapy and resolved solely with HSCT by day 24 post-transplant. The patient was 18 months at the time of bone marrow transplantation, and after receiving reduced intensity myeloablative conditioning, the rash has not recurred over the following 6 months.

## Conclusions

These cases emphasize the importance of heighted awareness for primary immunodeficiency in patients who present with frequent infections and who have unusual cutaneous findings that do not fit with common infant rashes. Our patients presented with a papulopustular dermatitis that was scattered throughout the extremities, refractory to routine therapies, and non-diagnostic on skin biopsy in conjunction with a characteristic infection associated with CGD. Afrough et al. described a somewhat similar vesiculopustular rash in a 24 day old male infant of the periorbita, genitalia, foot, and sacroiliac regions. Although these cases share a descriptive visual resemblance, the patient described by Afrouh et al. was quite sick and acquired multiple severe infections early in life, including osteomyelitis. Their patient's skin cultures were positive for *Staphylococcus aureus* and skin biopsies showed “necrotizing granulomatous tissue reaction with infectious etiology,” which aided in early diagnosis of CGD. In our first case, there was no identifiable skin infection or disease specific inflammation on skin biopsy to suggest a particular primary immunodeficiency. The resolution of the rash after HSCT in both cases highlights the relationship of this cutaneous finding with the underlying diagnosis of CGD. Specifically, our findings stress the need for considering CGD in a male infant presenting with generalized papulopustular rash in the right clinical context. Differential for generalized papulopustular rashes can include eczema, rosacea, acne, folliculitis, scabies, candida infection, cutaneous lupus, and other disorders. The need to assess infection history, other comorbidities, family history, and skin biopsies for rashes that are not improving with standard care, is essential to aid in diagnosis and work up of rare disease. Based on our findings, we would recommend consideration of dihydrorhodamine (DHR) analysis in children presenting with refractory papulopustular dermatitis with suggestive family and/or infectious history consistent with CGD. Further, it is important to note in such patients, HSCT led to resolution of these CGD associated symptoms, specifically papulopustular rash and colitis in our patients.

## Ethics Statement

The above was a case report involving human subjects who provided consent.

## Author Contributions

PR made significant contributions to the manuscript by drafting. MS was involved in drafting the manuscript and revising it critically for important intellectual content. PR and MS have given final approval of the version to be published.

### Conflict of Interest Statement

The authors declare that the research was conducted in the absence of any commercial or financial relationships that could be construed as a potential conflict of interest.

## References

[1] Agarwal S. Chronic granulomatous disease. J Clin Diagn Res. (2015) 9:SD01–02. 10.7860/JCDR/2015/12139.5945 26155526 PMC4484118

[2] Ben Abdallah Chabchoub R Turki H Mahfoudh A. [Systemic lupus erythematosus in a boy with chronic granulomatous disease: case report and review of the literature]. Arch Pediatr. (2014) 21:1364–6. 10.1016/j.arcped.2014.09.011 25445129

[3] Wolpowitz D Wise EM. Lupus-like rash of chronic granulomatous disease effectively treated with hydroxychloroquine. Cutis (2015) 95:E25–27. 26057516

[4] Afrough R Mohseni SS Sagheb S. An uncommon feature of chronic granulomatous disease in a neonate. Case Rep Infect Dis. (2016) 2016:5943783. 10.1155/2016/5943783 27872772 PMC5107835

[5] Vignesh P Gupta A Dogra S. Malar rash in a child with chronic granulomatous disease. J Allergy Clin Immunol Pract. (2017) 5:473–4. 10.1016/j.jaip.2016.11.013 28017630

